# Flesh Fly Maggot-Infected Abscess in a Burn Victim Patient in the United States Without a History of International Travel: A Case Report

**DOI:** 10.7759/cureus.39478

**Published:** 2023-05-25

**Authors:** Andy Aleman Espino, Erik Aleman Espino, Claudia Aleman Oliva, Gustavo Huaman, Ricardo Castrellon

**Affiliations:** 1 Kiran Patel College of Osteopathic Medicine, Nova Southeastern University, Fort Lauderdale, USA; 2 Department of Plastic Surgery, Larkin Community Hospital, Miami, USA; 3 Department of Plastic Surgery, Larkin Community Hopistal, Miami, USA

**Keywords:** infectious disease diagnosis, chronic wound, maggot, sacrophagidae, flesh fly, myiasis infestation, case report

## Abstract

This case report describes an uncommon occurrence of myiasis, specifically a maggot-infected abscess, in a patient with reduced skin sensitivity resulting from severe burns. Myiasis is the infestation of live animal tissue by fly larvae, and while it is primarily associated with tropical and subtropical regions, cases acquired within the United States are rare. The presented case involves a 70-year-old male who arrived at the emergency department with an intensely painful, non-healing wound in the left elbow. Upon examination, the wound was found to be infested with numerous live maggots, and subsequent investigations revealed the larvae to be of the flesh fly species (Sarcophagidae). The patient's history of reduced skin sensitivity, previous burn injuries, and exposure to outdoor environments, coupled with poor hygiene and homelessness, likely contributed to the infestation. This report emphasizes the importance of considering myiasis caused by flesh fly larvae even in non-travel-related cases within the United States. Early recognition and prompt treatment are vital to preventing complications and secondary infections. Healthcare providers should remain vigilant in identifying and managing myiasis, and patients with decreased skin sensation should be educated about the need for regular skin surveillance and the utilization of preventive measures to mitigate potential infestations.

## Introduction

This case report presents an unusual occurrence of a maggot-infected abscess in a patient with a history of decreased skin sensitivity caused by severe burns.

Myiasis is defined as a fly larva's infection of live animal tissue [1]. Flesh flies are common in most countries and are found in urban and rural communities [2]. The adult flies feed mostly on liquid substances and do not bite. However, their larvae can infect excrement, dead animals’ decaying flesh, and wounds [2]. 

Although the fly can carry diseases, such as leprosy bacilli, and a few cases have been reported causing myiasis in livestock, they pose little threat to humans [2,3]. When infection of live tissue occurs, hundreds of maggots colonize a single small area of skin, causing extensive lesions as they continue feeding on the resulting dead tissue [4].

On account of its ability to consume necrotic tissue, maggot debridement therapy has been used to treat infected or non-healing wounds for centuries, and it has gained popularity in recent years due to its effectiveness in treating chronic wounds [5]. However, an accidental infestation of wounds by the larvae has not been commonly described.

In the United States, most of the reported cases of myiasis are due to people who acquired the larva when traveling to tropical and subtropical areas of countries in Latin America and Africa. Cases of myiasis acquired in the United States without a history of previous travel are uncommon [1]. Today, we present a curious case of a wound infected with flesh fly larvae (Sarcophagidae) in a homeless patient with no prior travel history.

## Case presentation

A 70-year-old Hispanic male presented to our ER department in February 2023 with a chief complaint of intense pain in his left elbow for three days and a skin wound that was not healing. The patient attempted to clean and debride the wound but noticed that purulent drainage was still present afterward. On physical examination, the patient appeared well-developed but unkempt. He was alert and oriented to person, place, and time. The physical exam was remarkable only for a left arm (elbow) abscess. The patient's medical history was significant for hypertension, previous bilateral kidney surgery, and decreased sensitivity of the upper extremities due to healed severe burn lesions. On inspection, the affected area showed a sizeable erythematous wound with well-demarcated borders. The lesion was swollen and warm to the touch, with necrotic tissue and drainage. No areas of abrasions, lacerations, or ecchymosis were noted.

**Figure 1:**
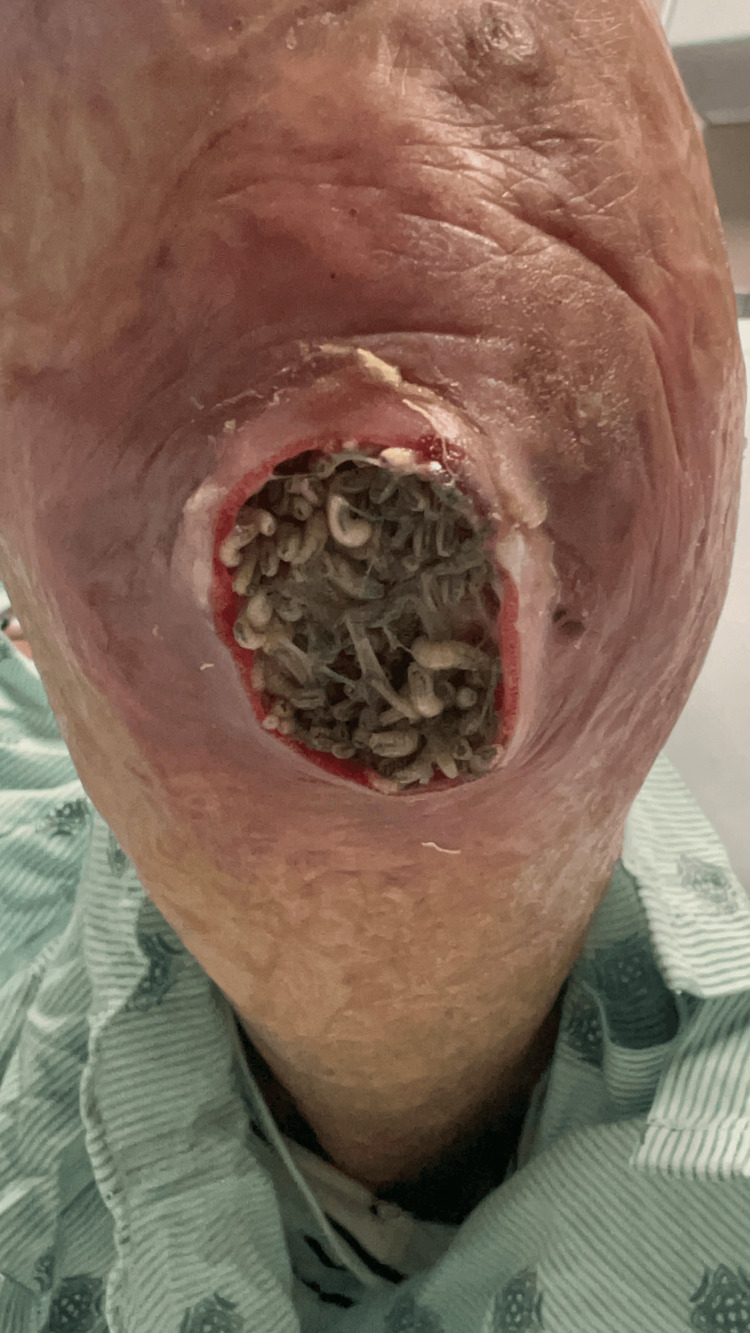
Uncovered wound with many live larvae

On the first attempt to uncover the wound, hundreds of live maggots (Figure 1) were easily seen with moderate amounts of foul-smelling purulent discharge. In a further interview with the patient, he communicated that he believed an insect bite might have caused the wound. He likes to work in his garden and does not use skin protection while doing so. He denied any fever, swelling, vomiting, recent trauma, or IV drug usage.

**Figure 2:**
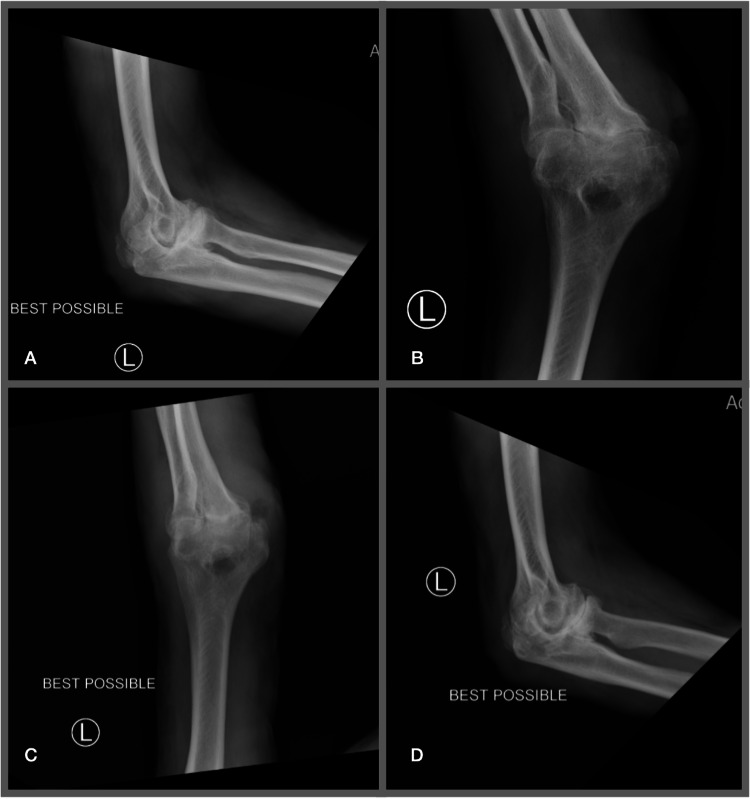
Left elbow X-ray (multiple views) A: Anterior-posterior view B: Anterior-posterior oblique with externally rotated arm C: Lateral view extended arm D: Lateral view flexed arm

The patient was started on vancomycin, piperacillin/tazobactam, and ivermectin, and the orthopedic department was consulted for possible left elbow bursitis or septic arthritis. A left elbow XR with 3+ views (Figure 2) was ordered and showed a soft tissue defect of the left elbow with soft tissue swelling, which may represent an ulcer, and thickening of the supracondylar humeral cortex, which may represent a previously healed fracture.

**Figure 3:**
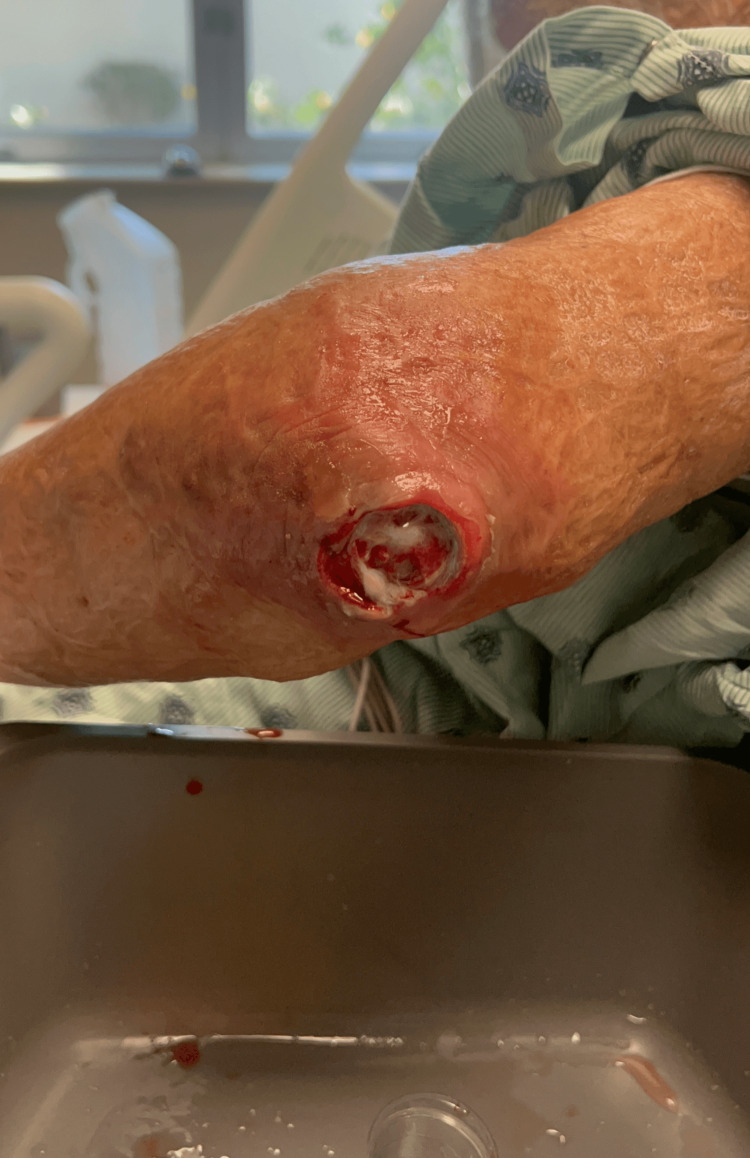
Left elbow wound after debridement and removal of larvae

The plastic surgery department was consulted for further management of the lesion. The team successfully removed the necrotic tissue and all visible larvae at the bedside with the help of a pulse irrigator (Figure 3). A tissue biopsy with larvae was taken and sent to the lab for proper identification. The patient was transferred to the care of the internal medicine team and the wound care team. They performed daily cleaning and packing of the lesion using Dakin’s solution and evaluated the patient for further medical workup. The pathology report confirmed that the maggots were flesh fly larvae (Sarcophagidae).

## Discussion

The Sarcophagidae family was initially found in temperate climates like the Arabian Peninsula and Japan, but nowadays, they inhabit a vast geographic distribution that includes Southern Florida and Central and South America [2].

The flesh-fly females are viviparous organisms. The eggs hatch inside the fly’s abdomen and are then deposited as larvae in the specific tissue. This mechanism gives the flesh fly larvae a significant advantage compared to their competitors when it comes to food and resource scavenging. The larvae can start feeding on tissue readily rather than waiting to hatch from the egg [6]. A delay in treatment allows the larvae to grow unrestricted, as each female can produce over a hundred eggs, and each is capable of producing more than 25 larvae at one time.

In the United States, most people with myiasis acquire it while traveling to areas of Africa and South America [1]. When it came to myiasis acquired in the United States, a series of studies by Francesconi et al. found that although numerous species of flies can cause myiasis, including Muscidae, Calliphoridae, and Sarcophagidae, about 87% of the cases were caused by flies belonging to the Calliphoridae family [7].

In the case of our patient, we speculate that a fly infected a pre-existing wound, probably while he was working in the garden, where the high temperatures and humidity characteristic of Florida weather make a suitable environment for these flies to thrive. The main predisposing factor leading to the advanced presentation of the infection in our patient is the loss of sensation in his elbows due to the extensive tissue damage from a previous burn accident. In addition, the lack of hygiene due to his homelessness status and his enjoyment of gardening, which exposed him to spending multiple hours lying with his elbows on the ground, are other contributing factors.

The management of our patient was focused on preventing further deterioration. The wound was debrided and irrigated, and broad-spectrum antibiotics covering gram-positives (including methicillin-resistant Staphylococcus aureus (MRSA)) and gram-negatives (including pseudomonas) were added in addition to the antiparasitic ivermectin, which has been successfully used in cases of myiasis [8,9].

Overall, this case report highlights the need for healthcare providers to be aware of the possibility of myiasis caused by flesh fly larvae, even in patients without a history of travel outside the United States. Early recognition and prompt treatment are crucial to prevent further complications, such as secondary infections or the spread of the infestation.

## Conclusions

In conclusion, this case report highlights an unusual occurrence of myiasis in a patient with a history of decreased skin sensitivity due to severe burns. The case illustrates the importance of maintaining a high index of suspicion for fly larvae infestation in wounds that are not healing properly, particularly in patients with a history of exposure to outdoor environments. Furthermore, this case underscores the importance of regular skin surveillance in patients with decreased sensation, as they may not feel or notice changes in their skin that could lead to complications, such as infection.

Preventive measures, such as using protective clothing and insect repellents, should be encouraged for patients with a history of outdoor exposure, particularly in areas where flesh flies are prevalent. Furthermore, healthcare providers should be educated on identifying and managing myiasis to ensure prompt diagnosis and treatment. Finally, patients with a history of decreased sensation should be advised to seek medical attention promptly if they notice any changes in their skin to prevent potential complications from developing.
